# Complete genome analysis of a novel recombinant isolate of pepper veinal mottle virus from mainland China

**DOI:** 10.1186/s12985-015-0419-9

**Published:** 2015-11-16

**Authors:** Songbai Zhang, Zibing Zhao, Limin Zheng, Jian Liu, Jing Peng, Fei Yan, Fan Li, Yan Xie, Zhaobang Cheng, Xuguo Zhou, Deyong Zhang, Yong Liu

**Affiliations:** Key Laboratory of Pest Management of Horticultural Crop of Hunan Province, Hunan Plant Protection Institute, Hunan Academy of Agricultural Science, Changsha, 410125 China; Longping Branch, Graduate College, Central South University, Changsha, 410125 China; Shaoyang University, Shaoyang, 422000 China; Virology and Biotechnology Institute, Zhejiang Academy of Agricultural Sciences, Hangzhou, 310021 China; Key Laboratory of Agricultural Biodiversity for Pest Management of China Education Ministry, Yunnan Agricultural University, Kunming, 650201 China; Institute of Biotechnology, Zhejiang University, Hangzhou, 310058 China; Institute of Plant Protection, Jiangsu Academy of Agricultural Sciences, Nanjing, 201014 China

## Abstract

**Background:**

*Pepper veinal mottle virus* (PVMV) was well established in Africa, and also reported infecting pepper (*Capsicum annuum* L) in Taiwan and India. However, there is not available of PVMV in mainland China. Here, the first complete genome sequence of PVMV isolated from pepper in mainland China was reported.

**Finding:**

The complete genomic sequence of isolate PVMV-HN isolated from pepper in mainland China is reported in this study. The genome of PVMV-HN is 9793 nucleotides (nt) excluding the poly (A) tail, shares 98-99 % nucleotide sequence identity with those two PVMV isolates from Ghana and Taiwan. Recombinant analysis showed that PVMV-HN probably represents a novel recombinant of PVMV. The phylogenetic relationship of PVMV-HN isolate to other PVMV isolates and other potyviruses based on genome or polyprotein sequence level and CP gene level, was also analyzed in this study.

**Conclusion:**

The current study will help to understand phylogenetic relationship of isolate PVMV-HN.

**Electronic supplementary material:**

The online version of this article (doi:10.1186/s12985-015-0419-9) contains supplementary material, which is available to authorized users.

## Background

Five *Potyvirus* spp. have been reported to infect pepper crops (*Capsicus* spp.) [1], of these, *Pepper veinal mottle virus* (PVMV), has been considered to be a major constraint to pepper production for yield and fruit quality reduction [2]. The virion of PVMV is flexuous filaments, enveloped a single molecule of linear, positive-sense, single-stranded ribonucleic acid (ssRNA), about 9.7-10 Kb in size, which has a poly (A) tract at the 3’ end, and its 5’ end covalently linked to the virus-encoded VPg protein [3, 4]. PVMV could form viral inclusion bodies and “pinwheels” in the cytoplasm of infected cells, which similar with other potyviruses [5], however, its serologically unrelated to other several pepper potyviruses, such as *Tobacco etch virus* (TEV) [6], *Potato virus Y* (PVY) [7] *and Chili veinal mottle virus* (ChiVMV) [8], and so on are extensively infecting pepper crops in China.

The first complete genome RNA sequence of PVMV was originally isolated from *Capsicus frutescens* in Ghana [9], and subsequently the other complete genome RNA sequence isolated from Taiwan have been deposited in GenBank (No. FJ617225). This virus is well documented of infected pepper crops in several countries in Africa [10, 11], and the phylogenetic relationship among different isolates with CP gene level has been extensively studied [9]. In Asia, this virus was reported infecting pepper crops in India and Taiwan [12]. However, as our best knowledge, there is no complete genome RNA sequence of PVMV infecting pepper crops in mainland China so far. In this study, the full genome sequence of a PVMV isolated from pepper (*Capsicum annuum*) in Hunan province, China was determined and compared to those of various PVMV isolates and other potyviruses.

### Viral material and sequence analysis

The viral symptoms of the pepper sample display mottle, yellowing and malformation on leaf, this pepper plant was sampled in a survey of pepper diseases in Hunan province, China in 2014. Total RNA infected pepper sample was extracted by an RNeasy mini kit (QIAGEN, Germany). Full-length cDNA was synthesized by using an oligo(dT)18 primer and TransScript® All-in-One First-Strand cDNA Synthesis SuperMix for PCR Kit (TransGen Biotech, Beijing,China) according to manufacturer’s introduction. The complete genome sequence of the isolate PVMV-HN was amplified from eight overlapping fragments using specific primers (Additional file 1: Table S1), and the 5’- and 3’-terminal ends of the genome was reconfirmed by 5’-RACE and 3’-RACE, respectively, as described previously [9]. PCR products were cloned into pEASY-T5 Vector (TransGen, Beijing, China) and sequenced by Sangon Biotechnology Co., Ltd. (Shanghai, China) using an ABI3730 automated DNA sequencer (Applied Biosystems, USA). Sequences were assembled using DNAMAN version 8 (Lynnon, Quebec, Canada). Sequences analysis and comparison of PVMV-HN to the reference sequences were performed using DNAStar 7.01 package (DNASTAR, Madison, USA). The complete RNA genome or polyprotein sequences and CP sequences of other PVMV isolates and other Potyviruses were downloaded from the National Center for Biotechnology Information (NCBI) database (http://www.ncbi.nlm.nih.gov/). The information of all used sequences was listed in Additional file 1: Table S2. Multiple nucleotide sequence alignments were performed with CLUST W, possible recombination events were evaluated by Recombination Detection Program (RDP3) [13] and a phylogenetic tree was reconstructed by the maximum-likelihood method using MEGA5 [14].

### Sequence properties

The complete genome of the PVMV-HN isolate comprises 9793 nucleotides (nt), with 194 nt at the 5’ non-translated region (NTR) and 373 nt at the 3’ NTR. Similar as other potyviruses, a large typical open reading frame (ORF) was identified as a polyprotein containing 3074 amino acids (aa) with an AUG start codon and UGA stop codon. PVM-HN shares 99 % nucleotide sequence identity with PVMV isolate of Ghana (DQ645484), and 98 % with PVMV isolate of Taiwan (FJ617225) with genome level, however, only 94 % nucleotide sequence identity with PVMV isolates (GQ918276 and GQ918274) from Mali with polyprotein gene level.

**Fig. 1:**
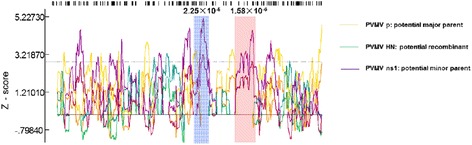
Recombination analysis showed two recombinant events of PVMV-HN (major parent: PVMV-p, Minor parent: PVMV-ns1) by RDP3

A search for possible recombination event was carried out using RDP3, there were three modules (Maxchi, Chimaera and SiScan) of RDP3 supported possible two recombination events between PVMV-HN with PVMV isolates from Ghana and Taiwan with high confidence (*p* < 2.25 × 10^−5^, Fig. 1). The sites of beginning and ending breakpoint were occurring at 3764 nt and 4146 nt, 4726 nt and 5275 nt, respectively. The potential major and minor parents were the PVMV-p (Ghana isolate) and PVMV-ns1 (Taiwan isolate). The existing two recombination events in PVMV-HN probably hinted it was a novel recombinant.

**Fig. 2:**
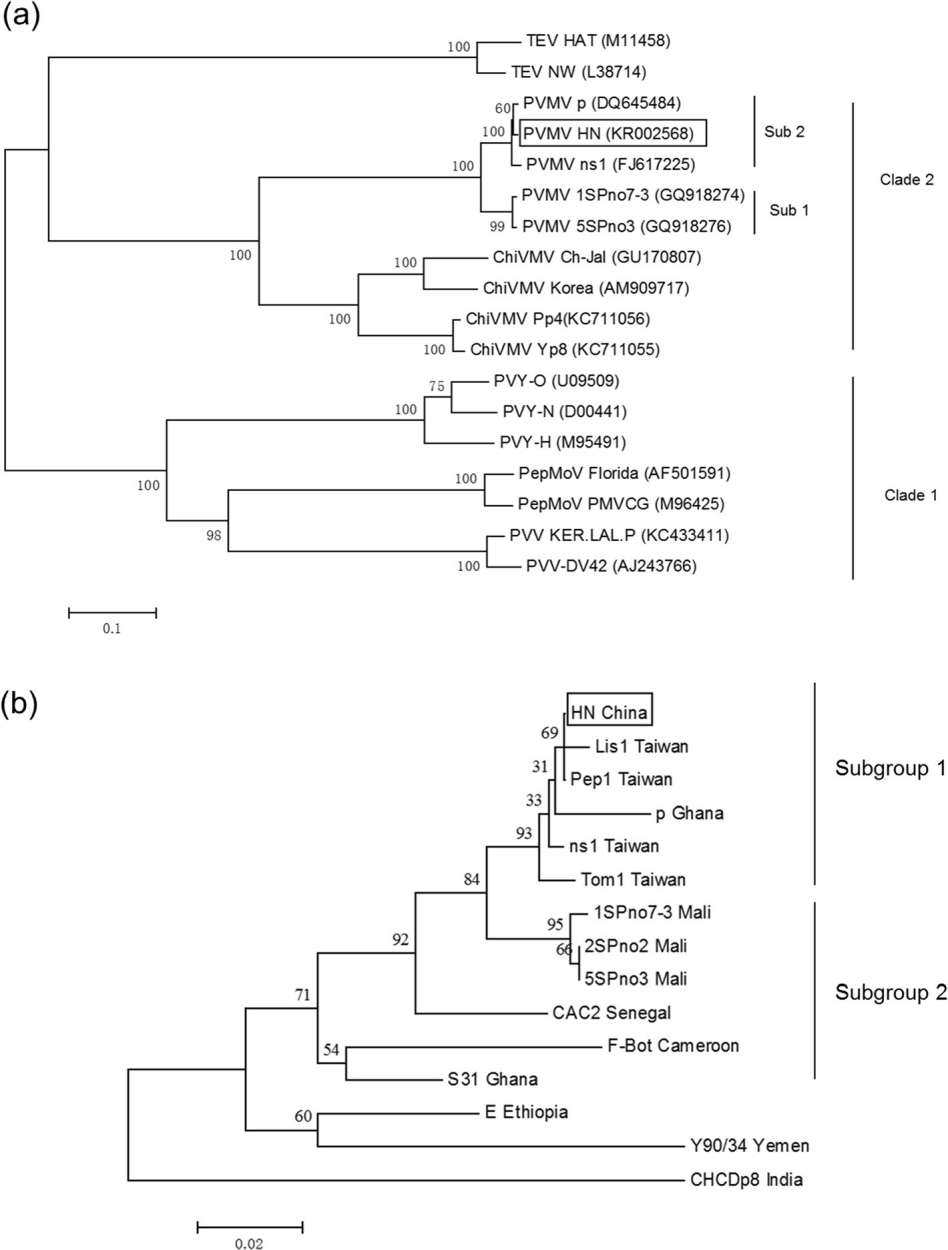
Phylogenetic analysis of PVMV-HN isolate (boxed) from China to other potyviruses based on complete genome or polyprotein nucleotide sequences (**a**) and to other PVMV isolates of different geographic origins based on CP gene nucleotide sequences (**b**). Bootstrap analysis was applied using 1 000 bootstrap samples. The scale bar represents the relative genetic distance. GenBank accession number: Pepper veinal mottle virus isolate p: FM202327, Pepper veinal mottle virus isolate ns1: FJ617225, Pepper veinal mottle virus isolate 1SPno7-3: GQ918274, Pepper veinal mottle virus isolate 5SPno3: GQ918276, Chilli veinal mottle virus isolate Ch-Ja1: GU170807, Chilli veinal mottle virus isolate Korea: AM909717, Chilli veinal mottle virus isolate Pp4: KC711056, Chilli veinal mottle virus isolate Yp8: KC711055, Tobacco etch virus isolate HAT: M11458, Tobacco etch virus isolate NW: L38714, Pepper mottle virus isolate Florida: AF501591, Pepper mottle virus isolate PMVCG: M96425, Potato virus V isolate KER.LAL.P: KC433411, Potato virus V isolate DV42: AJ243766, Potato virus Y isolate O : U09509, Potato virus Y isolate N: D00441, Potato virus Y isolate H: M95491

Phylogenetic analysis of PVMV-HN isolate from China to other potyviruses based on nucleotide sequences of complete genome or polyprotein gene level (Fig. 2a) and CP gene level (Fig. 2b) was built and two unique consensus trees topology were obtained. The two major clades were distinct existence well supported by bootstrap values associated to the branches of the tree (Fig. 2a). The first clade comprises of PVY, *Pepper mottle virus*, *Potato virus V,* and the second one includes PVMV and ChiVMV, shows close relationship of PVMV with ChiVMV, as mentioned by Moury et al. [1]. PVMV group was divided into two subgroups, the Africa subgroup and the East Asia subgroup. It is possible that the diversification of PVMV group was relevant with geography. Phylogenetic analysis of more PVMV isolates from different geographic origins based on nucleotide sequence of CP gene was also built and divided 12 PVMV isolates into two distinct major groups (Fig. 2b), reconfirmed that the diversification of PVMV group was relevant with geography, and PVMV-HN from China clustered in the East Asia subgroup. Additionally, phylogenetic analysis suggested that PVMV-HN likely originate from Taiwan and India as its closest evolution relationship (Fig. 2). Thus the recombination events (Fig. 1) were likely critical for PVMV-HN adaption and epidemic [15]. More sequences of PVMV isolates in China would help to understand phylogenetic relationship of PVMV in mainland China with that in other geography.

This study provided the first complete genome sequence about PVMV from mainland China and revealed it is probably a novel recombinant.

### Nucleotide sequence accession number

The complete genome sequence of the isolate of PVMV-HN is permanently available in the GenBank database under the accession number KR002568.

## Additional file

Additional file 1: Tabel S1. Primers used in this study. **Table S2.** Complete genome sequences quoted in this article. (DOC 64 kb)
