# Supplementary material for: Complete genome analysis of a novel recombinant isolate of pepper veinal mottle virus from mainland China
Source: Virol J. 2015 Nov 16;12:191. doi: 10.1186/s12985-015-0419-9 (PMC4647447; doi:10.1186/s12985-015-0419-9)
Supplement: Additional file 1: Tabel S1. — Primers used in this study. Table S2. Complete genome sequences quoted in this article. (DOC 64 kb) [file 12985_2015_419_MOESM1_ESM.doc]

**Supplementary Material**

Tabel S1 Primers used in this study

| Primer | Sequence（5’-3’） | Genome position |
| --- | --- | --- |
| PVMV1F | AAATAAAAACAACTCACACAGAC | 1-23 |
| PVMV1R | ACAAAGTTCCCATTCGCATCGAG | 1691-1713 |
| PVMV2F | TTCACAAGAATCGGGTTGAGTCAC | 1584-1607 |
| PVMV2R | CCTATCTGCCTCCTTCACGAGT | 3322-3343 |
| PVMV3F | AAATGCTCAGGAACTGTATGCGAT | 2755-2778 |
| PVMV3R | GTTGGCTCAAGCAATAGAACCC | 3969-3990 |
| PVMV4F | CCAAGTTGCCAGTGATATTAGCAG | 3859-3882 |
| PVMV4R | ACACAGTGCCACAACTTTTCGT | 5166-5187 |
| PVMV5F | GTCCGTGAATACAATCAATGC | 5075-5095 |
| PVMV5R | CATTTGTGCCAGATCACTCG | 6870-6889 |
| PVMV6F | ACCAAAACTACTTCGCATCCGTTC | 6846-6869 |
| PVMV6R | CTGTGTTCCTGCATTGACGTCT | 8711-8732 |
| PVMV7F | AGCAAATGGAAAAGCTCCGTA | 8450-8470 |
| PVMV7R | TGGCATATAAGACTTCTCCGA | 9141-9161 |
| PVMV8F | AATTAAGCCATTGATTGACCA | 9047-9067 |
| PVMV8R | AGCGCCAATTATGAAACCGC | 9776-9793 |

Table S2 Complete genome sequences quoted in this article

| Virus | Isolate | Country/Region | Host | Accession no. |
| --- | --- | --- | --- | --- |
| PVMV | P | Ghana | pepper | FM202327 |
| PVMV | ns1 | Taiwan | nightshade | FJ617225 |
| PVMV | HN | China | pepper | KR002568 |
| PVMV | 1SPno7-3 | Mali | Chilli pepper | GQ918274 |
| PVMV | 5SPno3 | Mali | tomato | GQ918276 |
| ChiVMV | Ch-Jal | India | Hot pepper | GU170807 |
| ChiVMV | Korea | South Korea | pepper | AM909717 |
| ChiVMV | Pp4 | China | pepper | KC711056 |
| ChiVMV | Yp8 | China | pepper | KC711055 |
| TEV | HAT | - | tobacco | M11458 |
| TEV | NW | - | - | L38714 |
| PepMoV | Florida | USA | pepper | AF501591 |
| PepMoV | PMVCG | USA | pepper | M96425 |
| PVV | KER.LAL.P | Iran | potato | KC433411 |
| PVV | DV42 | UK | - | AJ243766 |
| PVY | O | Canada | potato | U09509 |
| PVY | N | - | - | D00441 |
| PVY | H | - | - | M95491 |

PVMV: Pepper veinal mottle virus; ChiVMV: Chilli veinal mottle virus; TEV: Tobacco etch virus; PepMoV: Pepper mottle virus; PVV: Potato virus V; PVY: potato virus Y
